# Ebola Virus Disease in Pregnancy: Maternal–Fetal Outcomes, Vertical Transmission, and Implications for Vaccination—A Narrative Review

**DOI:** 10.3390/jcm15208169

**Published:** 2026-10-20

**Authors:** Khalid Nasralla Habeeballa Hashim, Hamid Mn. Mustafa, Ishag Adam

**Affiliations:** 1 Department of Obstetrics & Gynecology, College of Medicine, Qassim University, Buraydah 51452, Saudi Arabia; k.hashim@qu.edu.sa; 2 Department of Pharmacy Practice, College of Pharmacy, Qassim University, Buraydah 51452, Saudi Arabia; ha.mustafa@qu.edu.sa

**Keywords:** Ebola virus disease, pregnancy, maternal outcomes, fetal outcomes, neonatal outcomes, vertical transmission, placental infection, vaccination, rVSV ZEBOV, Ad26.ZEBOV/MVA-BN-Filo

## Abstract

**Background/Objectives:** Ebola virus disease (EVD) during pregnancy is associated with severe maternal illness and exceptionally poor fetal and neonatal outcomes. However, pregnancy-specific evidence remains limited by small cohorts, heterogeneous outbreak settings, and the historical exclusion of pregnant women from clinical research. This narrative review synthesizes evidence on maternal, fetal, and neonatal outcomes, placental involvement and vertical transmission, clinical management, and vaccination during pregnancy. **Methods:** PubMed, Scopus, and Google Scholar were searched from inception through May 2026 using predefined terms related to EVD, pregnancy, maternal and neonatal outcomes, placental infection, vertical transmission, and vaccination. WHO and CDC guidance documents and reference lists of relevant publications were also reviewed. Two reviewers independently screened records and assessed full texts. Data was extracted using a standardized template and independently verified by a second reviewer. A total of 55 publications met the inclusion criteria and were narratively synthesized. **Results:** Historical reports documented very high maternal mortality and pregnancy loss; however, more recent studies do not indicate that pregnancy independently increases maternal mortality among patients with EVD. A systematic review and meta-analysis estimated maternal mortality at 67.8% (95% CI 49.8–83.7), fetal loss at 76.9% (95% CI 45.0–98.3), and neonatal mortality at 98.5% (95% CI 84.9–100). Contemporary data from the 2018–2020 Democratic Republic of the Congo outbreak similarly found no increased maternal mortality associated with pregnancy, although fetal and perinatal outcomes remained poor. Evidence supports placental involvement in EVD, but placental viral RNA detection should be distinguished from confirmed fetal or neonatal infection. Pregnancy-specific vaccine evidence now includes a Phase 3 trial of Ad26.ZEBOV/MVA-BN-Filo in 2013 pregnant women, demonstrating favorable safety and immunogenicity profiles. **Conclusions:** EVD in pregnancy remains associated with substantial pregnancy loss and neonatal mortality despite improvements in maternal care. Important gaps persist regarding maternal–fetal transmission, long-term infant outcomes, and optimal management strategies, highlighting the need for further research.

## 1. Introduction

Ebola virus disease (EVD) is a severe zoonotic viral hemorrhagic fever caused by members of the genus *Ebolavirus* within the family *Filoviridae* [1]. The disease is characterized by rapid clinical deterioration, multisystem involvement, and high case-fatality rates, particularly in settings with limited healthcare capacity. Historically, mortality has ranged from 25% to 90%, depending on viral species and access to supportive care, making EVD one of the most lethal emerging infectious diseases [2].

Filovirus outbreaks were first recognized in 1967 during an epidemic of Marburg virus disease in Germany and the former Yugoslavia [3]. EVD itself was first identified in 1976 during simultaneous outbreaks in southern Sudan and northern Zaire (now the Democratic Republic of the Congo, DRC). Since then, recurrent outbreaks have occurred across sub-Saharan Africa, causing substantial morbidity and mortality and disrupting fragile health systems. Major outbreaks since 1976 are summarized in Table 1.

The largest recorded EVD epidemic occurred in West Africa between 2014 and 2016, resulting in more than 28,000 cases and over 11,000 deaths [4]. This epidemic highlighted the consequences of delayed detection, weak surveillance systems, and inadequate outbreak preparedness [5].

At present, neither the WHO nor other international public-health agencies provide quantitative forecasts of the future global frequency of Ebola outbreaks. However, modeling studies suggest that Ebola virus spillover events will likely continue to occur and may increase in some regions because of environmental change, forest fragmentation, and increasing human–wildlife interactions. Current forecasting efforts are primarily directed toward identifying geographic areas of elevated spillover risk and projecting the short-term course of ongoing outbreaks rather than predicting long-term outbreak frequency [6,7,8].

Pregnant women represent a uniquely vulnerable population during EVD outbreaks. Physiological adaptations of pregnancy, including altered immunity, increased cardiovascular demand, and hypercoagulability, may worsen disease severity and increase the risk of complications [9,10]. Although recent evidence suggests that maternal case-fatality rates may be like those of non-pregnant women when high-quality supportive care is available [11], pregnancy remains strongly associated with adverse obstetric outcomes. Miscarriage, intrauterine fetal demise, stillbirth, preterm birth, and neonatal death occur at exceptionally high rates, and fetal survival has historically been rare. Furthermore, placental tissue, fetal membranes, amniotic fluid, and other pregnancy-related fluids contain high viral loads, creating substantial risks of transmission to healthcare workers and birth attendants [12].

Growing virological and pathological evidence demonstrates that the Ebola virus can infect the fetoplacental unit and cross the maternal–fetal interface, supporting the occurrence of in utero transmission [12,13]. These findings have important implications for fetal outcomes, infection control, and clinical management.

Vaccine development efforts began in the late 1990s but accelerated dramatically following the 2014–2016 West African epidemic. The recombinant vesicular stomatitis virus–Zaire Ebola virus vaccine (rVSV-ZEBOV; Ervebo^®^) demonstrated high effectiveness and became the first licensed Ebola vaccine in 2019, recommended for outbreaks caused by *Zaire ebolavirus* [14]. However, no licensed vaccines exist for other species such as *Bundibugyo ebolavirus*, which continues to cause outbreaks [15]. Pregnant women were historically excluded from vaccine trials due to concerns about fetal safety, resulting in major evidence gaps. More recently, ethical frameworks have encouraged their inclusion in outbreak-related research, generating emerging safety and immunogenicity data [16].

At the time of writing, an active outbreak of *Bundibugyo ebolavirus* is ongoing in eastern DRC, with confirmed cross-border transmission into Uganda. In May 2026, the World Health Organization (WHO) declared this outbreak a Public Health Emergency of International Concern due to sustained transmission, regional spread, and the absence of licensed vaccines or specific therapies for this species [7]. This event underscores the urgent need for improved preparedness, maternal health protection, and effective preventive strategies.

Given the persistent threat of Ebola outbreaks, the disproportionate burden of disease among pregnant women and their infants, and recent advances in therapeutics and vaccination, a comprehensive synthesis of current evidence is warranted. This review summarizes maternal, fetal, and neonatal outcomes of EVD during pregnancy; evaluates evidence for placental infection and vertical transmission; and critically examines emerging data on Ebola vaccination in pregnancy. The review also highlights implications for clinical management, outbreak preparedness, and future research priorities.

## 2. Materials and Methods

### 2.1. Review Design and Rationale

This study was conducted as a narrative review because the evidence concerning EVD in pregnancy is clinically important but methodologically heterogeneous, comprising historical outbreak reports, case reports and series, observational cohorts, pathological and virological studies, systematic reviews, vaccine studies, surveillance reports, and clinical guidance. The review was structured in accordance with the Scale for the Assessment of Narrative Review Articles (SANRA) to improve transparency of the search, evidence selection, and critical synthesis.

### 2.2. Information Sources and Search Strategy

PubMed, Scopus, and Google Scholar were searched from database inception through May 2026. Search concepts covered Ebola virus disease, pregnancy, maternal outcomes, fetal and neonatal outcomes, placental infection, vertical transmission, and Ebola vaccination. WHO and CDC guidance documents were searched separately, and reference lists of relevant reviews and primary studies were examined for additional records. Google Scholar was used as a supplementary source. Because a prespecified stopping rule had not been established prospectively, this represents a limitation of the search methodology and is acknowledged accordingly. The exact database-specific search strings, date of the last search, filters applied, and Google Scholar screening procedure are provided in Supplementary Table S1.

### 2.3. Eligibility Criteria

Eligible publications reported clinical, epidemiological, obstetric, neonatal, virological, pathological, therapeutic, or vaccination data relevant to EVD in pregnancy. Primary human evidence included case reports, case series, outbreak investigations, observational cohorts, and clinical trials. Systematic or narrative reviews, surveillance reports, and authoritative guidance documents were retained as supporting sources but were distinguished from primary studies in the evidence synthesis. English-language publications were included. Animal or laboratory studies were considered only when directly relevant to placental pathogenesis or mechanisms of maternal–fetal transmission. Publications without pregnancy-specific information or without direct relevance to the review questions were excluded.

### 2.4. Study Selection, Deduplication, and Record Accounting

Records from bibliographic databases were imported into [Mendeley Reference Manager v2.149.0]. Duplicate records were removed using Mendeley, followed by a manual checking procedure. Two reviewers independently screened titles and abstracts and assessed potentially eligible full texts, resolving disagreements through discussion and consensus. The number of records identified was 653 (detailed in Supplementary Table S1. Database search and study-selection reporting); records remaining after deduplication are 112 articles, whose abstracts were screened for relevance; full-text assessed publications were 60. Full-text articles excluded were n = 5 (duplicate/overlapping cohort publications (n = 3), insufficient pregnancy-specific data (n = 1), and did not meet inclusion criteria (n = 1)). The final primary studies contributing pregnancy-specific clinical data were 55. Multiple articles describing overlapping patients or outbreak cohorts were cross-checked and were not treated as independent patients when calculating or comparing clinical outcomes.

### 2.5. Data Extraction and Verification

Data were extracted using a standardized template capturing outbreak period and country, Ebola virus species, study design, number of pregnant women, gestational age or trimester, diagnostic method, treatment, maternal outcome, pregnancy loss, live birth, neonatal outcome, follow-up duration, placental or fetal virology/pathology, vaccination exposure, and major methodological limitations.

### 2.6. Outcome Definitions and Unit of Analysis

Outcome definitions varied across the included studies. Miscarriage was defined as pregnancy loss before fetal viability, fetal death as death before complete expulsion or extraction from the mother, stillbirth as fetal death occurring after the gestational-age threshold used to define viability, and neonatal death as death within the first 28 days of life. Because many studies did not explicitly report outcome definitions and reporting standards differed across outbreaks, the terminology used by the original authors was retained whenever possible. Outcomes were classified as “definition not reported” when explicit criteria were unavailable. Outcome terminology was interpreted according to the definitions reported by each primary study. Such variability should be considered when interpreting and comparing pregnancy outcomes across studies. Miscarriage or spontaneous abortion, fetal death, stillbirth, live birth, and neonatal death were recorded separately when the source permitted. Because gestational-age thresholds were not consistently reported across outbreaks and historical publications, no uniform retrospective threshold was imposed. For clinical outcome synthesis, the pregnancy or mother–infant dyad was treated as the unit of interest wherever individual cases could be identified. Potentially overlapping reports were flagged and not double-counted.

### 2.7. Evidence Synthesis

Because study designs, outbreak settings, virus species, diagnostic approaches, treatment availability, and outcome definitions were heterogeneous, no new meta-analysis was undertaken. The review instead provides a quantitative and critical study-level narrative synthesis. Published pooled estimates are cited as contextual evidence but are not combined with overlapping primary-study data.

## 3. Results

### 3.1. Virology and Transmission of Ebola Virus in Pregnancy

Ebola virus disease (EVD) is caused by enveloped, negative-sense, single-stranded RNA viruses of the genus *Ebolavirus* within the family *Filoviridae*. Several species have been identified, including *Zaire ebolavirus*, *Sudan ebolavirus*, and *Bundibugyo ebolavirus*, which differ in virulence, geographic distribution, and epidemic potential [17,18]. *Zaire ebolavirus* is the most virulent and has caused most large outbreaks, including the 2014–2016 West African epidemic.

Human infection typically follows zoonotic spillover from wildlife—particularly fruit bats, the presumed natural reservoir—and subsequent human-to-human transmission through direct contact with infected blood or body fluids [17]. Viral loads rise rapidly during illness and peak in late disease and after death, making caregiving, healthcare exposure, and traditional burial practices major drivers of transmission [19].

Pregnancy introduces additional transmission challenges. High viral concentrations have been documented in the placenta, amniotic fluid, fetal membranes, umbilical cord blood, and products of conception [13,17]. These tissues may remain infectious even after maternal recovery, functioning as viral reservoirs and posing substantial risks to obstetric care providers. Labor, delivery, miscarriage management, and handling of stillbirths are particularly high-risk events due to exposure to large volumes of infectious fluids [20,21,22].

Evidence of Ebola virus RNA or antigen in placental tissues supports infection of the fetoplacental unit and provides biological plausibility for maternal–fetal transmission. Nevertheless, the presence of viral material in placental tissue alone does not prove productive fetal infection, and distinctions should be made between placental infection, fetal infection, and neonatal infection when interpreting transmission pathways [21,23]. Table 2 compares major Ebola virus species with respect to virulence, outbreak history, and implications for pregnancy. The absence of licensed vaccines for *Bundibugyo ebolavirus* remains a critical gap in protecting pregnant women during outbreaks.

### 3.2. Pathophysiology and Immunology of EVD During Pregnancy

EVD pathogenesis is characterized by widespread viral dissemination, profound immune dysregulation, endothelial injury, and multiorgan failure. Following entry through mucosal surfaces or broken skin, the Ebola virus infects monocytes, macrophages, and dendritic cells, which disseminate the virus to multiple organs, including the liver, spleen, adrenal glands, kidneys, and reproductive tract [24,25].

A hallmark of severe disease is the uncontrolled release of pro-inflammatory cytokines—TNF-α, IL-6, IL-8, and others—resulting in a “cytokine storm” that drives endothelial dysfunction, vascular leakage, hypotension, and shock [26,27]. Concurrently, impaired dendritic cell function and extensive lymphocyte apoptosis contribute to profound immunosuppression.

Coagulation abnormalities are common. Thrombocytopenia and disseminated intravascular coagulation (DIC) may develop, contributing to bleeding manifestations and multiorgan dysfunction [28]. Hepatic injury, adrenal insufficiency, and acute kidney injury further exacerbate clinical deterioration [29,30].

Pregnancy-specific physiological adaptations, including altered innate and adaptive immunity, may influence susceptibility to severe disease [31,32]. Although recent evidence suggests that maternal case fatality rates may not be significantly higher than in non-pregnant women when high-quality supportive care is available [33], pregnancy remains strongly associated with adverse obstetric and perinatal outcomes.

The placenta plays a central role in disease pathogenesis. Placental infection is associated with inflammation, vascular injury, and disruption of maternal–fetal exchange, contributing to fetal hypoxia, growth restriction, and fetal demise. The mechanisms of placental infection and maternal–fetal transmission remain incompletely understood [34,35].

Host genetic factors may contribute to individual variation in susceptibility to Ebola virus infection and disease severity, although evidence in pregnant women remains extremely limited. Studies investigating human immunogenetic variation have identified associations between specific *killer-cell immunoglobulin-like receptor* (*KIR*) genes and *human leukocyte antigen* (*HLA*) class I alleles and clinical outcomes of Ebola virus disease (EVD), suggesting that innate and adaptive immune responses may influence survival. KIR2DL2 was more frequently observed among survivors, whereas *KIR2DL5* and *KIR2DS4-003* were associated with fatal outcomes [36]. Additionally, genetic variation in the *Niemann-Pick C1* (*NPC1*) gene, which encodes the cellular receptor required for Ebola virus entry, has been shown to alter cellular susceptibility to filovirus infection, providing a biologically plausible mechanism for host-dependent differences in infection risk [37]. However, no large human genome-wide association studies (GWAS) have been conducted for EVD, and no studies have specifically evaluated the influence of maternal or fetal genetic variation on pregnancy-related Ebola outcomes. Consequently, the potential role of maternal and fetal genetics in susceptibility, vertical transmission, fetal survival, and neonatal outcomes remains largely unexplored and represents an important priority for future research.

### 3.3. Clinical Presentation and Diagnosis of EVD in Pregnancy

The clinical presentation of EVD in pregnancy is broadly similar to that in non-pregnant adults but may be more difficult to recognize due to overlap with common obstetric conditions. The incubation period ranges from 2 to 21 days, with symptoms typically developing 8–10 days after exposure [10,38].

Early symptoms are non-specific and include fever, severe headache, myalgia, arthralgia, sore throat, and malaise [2]. As the disease progresses, gastrointestinal symptoms—nausea, vomiting, abdominal pain, anorexia, and profuse watery diarrhea—often dominate and may lead to severe dehydration, hypovolemia, electrolyte disturbances, and shock. These complications can severely compromise uteroplacental perfusion, contributing to fetal distress and fetal demise [2,10].

Although historically classified as a viral hemorrhagic fever, overt bleeding occurs in only a minority of cases. When present, hemorrhagic manifestations—petechiae, ecchymoses, gingival bleeding, epistaxis, gastrointestinal bleeding, hematuria—typically indicate advanced disease and are associated with thrombocytopenia and DIC [39,40].

Pregnancy-specific complications are common. Vaginal bleeding, miscarriage, intrauterine fetal death, stillbirth, preterm labor, and premature rupture of membranes have all been reported [10,41]. In some cases, obstetric complications may be the presenting feature of EVD.

Laboratory abnormalities include leukopenia followed by neutrophilia, thrombocytopenia, elevated liver enzymes, electrolyte disturbances, renal impairment, and coagulation abnormalities [42,43,44]. However, none are diagnostic.

Definitive diagnosis relies on real-time reverse transcription polymerase chain reaction (RT-PCR), the gold standard for detecting Ebola virus RNA [42,43,45]. During pregnancy, special diagnostic considerations apply because viral RNA may be present in placental tissue, fetal membranes, amniotic fluid, cord blood, and products of conception [33,35,46].

### 3.4. Maternal Outcomes of EVD in Pregnancy

Historical reports described extremely high maternal mortality, but these observations arose from small outbreak cohorts and should not be interpreted as evidence that pregnancy itself independently increases the risk of death. In the 1995 Kikwit outbreak, Mupapa et al. described 15 pregnant women among 105 women hospitalized with Ebola hemorrhagic fever; only one pregnant woman survived, and severe bleeding was reported in all 15 [10]. The study was retrospective; pregnancy diagnosis relied on history and clinical examination, and available supportive care differed substantially from contemporary Ebola treatment.

Evidence synthesized across outbreaks suggests lower and more heterogeneous maternal mortality. Foeller et al. reported 197 deaths among 274 pregnant women with EVD (72%) in a systematic review of 52 included studies and concluded that mortality among pregnant women was not higher than in the overall EVD population [33]. A subsequent systematic review and meta-analysis of 28 studies estimated an absolute maternal mortality risk of 67.8% (95% CI 49.8–83.7), with substantial heterogeneity (I^2^ = 85%); the pooled relative risk comparing pregnant and nonpregnant patients was 1.18 (95% CI 0.59–2.35) [47]. These pooled estimates should be interpreted cautiously because they combine outbreaks differing in virus species, case ascertainment, supportive-care capacity, and availability of therapeutics.

Contemporary data further illustrate the effect of clinical context. Philpott et al. examined 27 pregnant women admitted to the Mangina Ebola Treatment Center during the 2018–2020 DRC outbreak. In a propensity-score-matched analysis incorporating age, initial Ebola viral load, vaccination status, and investigational therapeutic exposure, pregnancy was not associated with increased mortality compared with nonpregnant women of reproductive age (RR 1.0, 95% CI 0.58–1.72) [48]. Accordingly, the current evidence supports a distinction between the high absolute severity of EVD and the more uncertain question of whether pregnancy independently increases maternal mortality.

### 3.5. Pregnancy, Fetal, and Neonatal Outcomes

Pregnancy and neonatal outcomes remain poor even where maternal survival has improved. The meta-analysis by Kayem et al. estimated an absolute fetal-loss risk of 76.9% (95% CI 45.0–98.3; I^2^ = 96%) and neonatal mortality of 98.5% (95% CI 84.9–100) among the available studies [47]. The very high heterogeneity for fetal loss indicates that a single pooled percentage should not be interpreted as a universal risk applicable to every outbreak or treatment setting.

In the Mangina cohort, at least 58% (11/19) of surviving mothers experienced pregnancy loss, while 16% (3/19) were discharged with a viable pregnancy. Two live births were reported, of whom one infant survived [48]. Earlier case reports and small series also document pregnancy loss despite maternal recovery. For example, Baggi et al. reported two pregnant women with confirmed EVD in Guinea in 2014 who survived after treatment in an Ebola treatment center; both underwent induced vaginal delivery [41]. These small reports are clinically informative but cannot provide stable incidence estimates.

Differences between studies likely reflect, at least in part, differences in sample size, gestational age, viral species, disease severity, diagnostic certainty, access to supportive care, obstetric resources, vaccination, and disease-directed treatment.

### 3.6. Placental Infection and Evidence for Vertical Transmission

Evidence of Ebola virus involvement of the fetoplacental unit must be interpreted according to the biological compartment tested and the diagnostic method used. Detection of Ebola viral RNA by RT-PCR in placental tissue demonstrates the presence of viral genetic material, but this finding alone does not establish productive viral replication or independently confirm fetal infection. More direct evidence of placental cellular infection is provided by histopathology, immunohistochemistry, or other methods that localize viral antigen within defined placental cell types.

Muehlenbachs et al. described placental pathology in pregnancy-associated EVD caused by Sudan virus and Bundibugyo virus. Ebola virus antigen was demonstrated by immunohistochemistry in syncytiotrophoblast and maternal placental mononuclear cells; antigen was not identified in fetal placental stromal cells or fetal organs in the cases examined. The authors interpreted trophoblast infection as a possible mechanism of transplacental transmission [35]. These findings support active placental infection but also illustrate why placental infection should not be treated as synonymous with confirmed fetal infection.

Evidence supporting fetal infection becomes stronger when viral material or antigen is demonstrated in fetal tissues or fetal compartments using specimens in which maternal contamination is unlikely. Conversely, neonatal infection detected after delivery should not automatically be classified as in-utero transmission because antenatal, intrapartum, and postnatal exposure are biologically distinct possibilities. A reported case of maternal recovery with loss of the fetus showed maternal blood becoming negative for Ebola viral RNA while vaginal secretions, placental tissue, umbilical cord, and neonatal buccal swabs remained RNA-positive, demonstrating persistence in pregnancy-associated compartments but not, by RT-PCR alone, productive infection in every positive tissue [49].

Accordingly, this review uses four distinct terms: (1) placental viral RNA detection, (2) evidence of active placental infection, (3) evidence of fetal infection, and (4) neonatal infection. This hierarchy avoids overstating the inference that can be drawn from a positive molecular result and separates biological evidence for transplacental infection from infection acquired around the time of delivery.

Pregnant women with EVD are at high risk of severe complications. Earlier outbreaks reported near-universal maternal mortality [50,51], although more recent data suggest that maternal case-fatality rates may approximate those of non-pregnant women when high-quality supportive care is available [11,33].

Maternal death typically results from a combination of hypovolemia, electrolyte disturbances, coagulopathy, secondary infections, and multiorgan failure. Persistent vomiting and diarrhea contribute to profound fluid losses, while hepatic and renal dysfunction and cardiovascular collapse are major determinants of mortality [30,41].

Hemorrhagic complications are a major source of morbidity. Pregnant women may experience vaginal bleeding associated with miscarriage, stillbirth, or postpartum hemorrhage. Thrombocytopenia and DIC further increase the risk of severe obstetric hemorrhage, which is often difficult to manage in outbreak settings with limited access to blood products and surgical services [20,41]. Table 3 summarizes the major maternal outcomes reported across Ebola virus disease outbreaks. Consistent with previous reports, pregnant women experience high rates of severe complications, including hypovolemia, coagulopathy, hemorrhage, and multiorgan failure. Although maternal survival has improved in recent outbreaks, morbidity remains substantial.

The characteristics of the principal studies contributing pregnancy-specific clinical data are summarized in Table 4. The included studies span multiple Ebola outbreaks, viral species, and clinical settings, highlighting substantial heterogeneity in study design, sample size, treatment availability, and outcome reporting.

### 3.7. Fetal and Neonatal Outcomes

As shown in Table 4, adverse pregnancy outcomes were reported across all major outbreak settings, although the magnitude of risk varied according to study design, clinical setting, and availability of supportive care and investigational therapies.

Fetal and neonatal outcomes in EVD are exceptionally poor. Miscarriage, intrauterine fetal demise, and stillbirth occur at extremely high rates across all trimesters. Neonatal survival is rare, even when mothers survive the acute illness. High viral loads in placental and fetal tissues, combined with placental inflammation and vascular injury, contribute to these outcomes. Fetal and neonatal outcomes are uniformly poor across outbreaks, as summarized in Table 5. Miscarriage, intrauterine fetal demise, and stillbirth occur at exceptionally high frequencies, and neonatal survival remains rare even when maternal recovery occurs.

The study-level data presented in Table 6 demonstrate that improvements in maternal survival have not consistently translated into improved fetal or neonatal outcomes.

To place findings from individual outbreak reports into a broader context, major systematic reviews and meta-analyses are summarized in Table 6. These syntheses provide pooled estimates of maternal mortality, fetal loss, and neonatal mortality while also illustrating the substantial heterogeneity of the available evidence.

The pooled estimates summarized in Table 6 should be interpreted cautiously because they combine evidence originating from different Ebola virus species, outbreak periods, healthcare settings, and treatment eras. Nevertheless, they consistently demonstrate that fetal and neonatal outcomes remain considerably worse than maternal outcomes, even in more recent cohorts.

### 3.8. Placental Infection and Vertical Transmission

Multiple studies have demonstrated Ebola virus in placental tissue, amniotic fluid, fetal membranes, cord blood, and fetal tissues [13,35]. These findings provide strong evidence for vertical transmission and explain the high frequency of fetal loss. The placenta may act as an immune-privileged reservoir, remaining infected even after maternal recovery. Table 7 summarizes virological evidence supporting vertical transmission. Detection of Ebola virus in placental tissue, amniotic fluid, cord blood, and fetal tissues provides strong biological support for in utero infection and explains the high frequency of fetal loss.

### 3.9. Vaccination in Pregnancy

Evidence concerning Ebola vaccination during pregnancy should be assessed in three separate domains: (1) efficacy or effectiveness against disease caused by the target virus; (2) pregnancy-specific safety and immunogenicity; and (3) recommendations for use during pregnancy in a particular outbreak. Evidence in one domain does not automatically establish another, and evidence for a vaccine directed against Zaire ebolavirus should not be extrapolated without evidence to other Ebola virus species.

The rVSVΔG-ZEBOV-GP vaccine has demonstrated protection against Ebola virus disease in outbreak settings, but the original efficacy trials largely excluded pregnancy. Pregnancy-specific evidence therefore initially came from inadvertent or peri-conception exposures. In the Sierra Leone STRIVE analysis, 84 women were inadvertently vaccinated in early pregnancy or became pregnant within 60 days of vaccination or enrolment. Pregnancy loss occurred in 14/31 (45%) immediately vaccinated women versus 11/33 (33%) unvaccinated women with known contemporaneous pregnancies (RR 1.35, 95% CI 0.73–2.52); no congenital anomalies were identified among 44 live-born infants examined [52,53]. These observational data are informative but limited by the small number of exposed pregnancies and the fact that pregnancy was an exclusion criterion in the parent trial.

Pregnancy-specific randomized evidence is now available for the heterologous Ad26.ZEBOV/MVA-BN-Filo regimen. Nyombayire et al. randomized 2013 healthy pregnant women in Rwanda to vaccination during pregnancy or postpartum. Adverse outcomes of interest occurred in 5.2% of women vaccinated during pregnancy and 7.3% of women in the comparator group; corresponding infant outcomes of interest occurred in 26.0% and 25.6%, respectively. The study reported no safety concerns regarding adverse maternal/fetal or neonatal/infant outcomes. Ebola virus glycoprotein-specific binding antibody responses were sustained in at least 90% of women at one year after dose 1, and antibodies were detected in cord blood and infant serum, supporting passive transfer [54]. This trial substantially strengthens pregnancy-specific safety and immunogenicity evidence for this regimen, but it should not be described as a clinical-efficacy trial demonstrating prevention of maternal EVD during pregnancy.

Species specificity remains essential. Evidence supporting licensed or established vaccine protection against Zaire ebolavirus should not be generalized to Bundibugyo virus. Candidate approaches for non-Zaire Ebola viruses should be described according to their regulatory and investigational status at the time of the review, using current WHO guidance rather than assuming cross-protection, Table 8.

### 3.10. Clinical Management Considerations

#### 3.10.1. Triage, Diagnosis, and Isolation

Pregnant patients with compatible symptoms and epidemiological exposure require prompt assessment using the same basic principles of EVD recognition and infection prevention applied to other patients, while accounting for overlap between EVD symptoms and pregnancy-related complications. WHO emphasizes that pregnant women should receive equitable care and should not be excluded from appropriate EVD management because of pregnancy [46]. Suspected or confirmed cases require care in an environment capable of appropriate isolation, diagnostic testing, infection prevention and control, and obstetric assessment. Because miscarriage, labor, fetal death, or hemorrhage can complicate EVD, obstetric expertise should be integrated with infectious-disease and outbreak-response care.

#### 3.10.2. Optimized Supportive and Disease-Directed Care

WHO recommends optimized supportive care for all pregnant women with acute EVD, although the pregnancy-specific evidence supporting many components is of very low quality [46]. Optimized care includes repeated assessment, fluid resuscitation, electrolyte monitoring and correction, glucose monitoring and management, treatment of potential co-infections, nutritional support, symptomatic treatment, and prevention and management of complications [46]. Where intensive-care resources are available, organ support should be considered according to maternal clinical need. Pregnancy alone should not be used as a reason to provide less intensive supportive care.

#### 3.10.3. Obstetric Hemorrhage, Pregnancy Loss, and Delivery

Obstetric management requires simultaneous attention to maternal stabilization, obstetric indication, fetal status or viability, and occupational exposure risk. Hemorrhage may be compounded by thrombocytopenia and coagulopathy. Decisions regarding induction, operative procedures, and mode or timing of delivery should therefore be individualized rather than dictated solely by EVD status. Historical and contemporary reports show that vaginal delivery or induction has been used in selected patients, but small case series cannot establish a universally preferred delivery strategy [41]. Procedures involving blood, amniotic fluid, placental tissue, fetal membranes, or products of conception require stringent infection-prevention planning because pregnancy-associated tissues and fluids may remain RNA-positive after maternal blood becomes negative [33,49].

#### 3.10.4. Neonatal Management

Infants born to women with acute or recently resolved EVD require coordinated neonatal, infectious-disease, laboratory, infection-control, and obstetric management. Diagnostic interpretation should consider the timing and type of neonatal specimen. A positive neonatal test confirms infection when diagnostic criteria are met but does not by itself determine whether acquisition occurred in utero, during delivery, or after birth. The evidence base for neonatal treatment and long-term outcomes remains sparse [33].

#### 3.10.5. Breastfeeding and Viral Persistence

Breastfeeding requires specific risk assessment because Ebola virus RNA has been detected in breast milk, and systematic-review evidence supports a potential transmission risk from breast milk and pregnancy-related fluids [33]. WHO guidance addresses breastfeeding women as a distinct clinical group and emphasizes management across exposure, acute illness, and recovery [46]. Viral RNA can persist in pregnancy-associated compartments after maternal blood clearance; Foeller et al. reported detection in amniotic fluid for up to 32 days after maternal clearance of Ebola virus from blood [33]. Molecular persistence, however, should not automatically be equated with proven infectivity of every RNA-positive specimen.

#### 3.10.6. Multidisciplinary Decision-Making and Uncertainty

Pregnancy-specific management evidence remains limited. Important uncertainties include the optimal timing and mode of delivery, fetal surveillance, thresholds for invasive obstetric procedures, management of severe obstetric hemorrhage in resource-limited Ebola treatment units, neonatal treatment, and the infectious significance of persistent viral RNA in gestational tissues. Decisions should therefore be individualized and multidisciplinary, incorporating maternal condition, gestational age and fetal status, available obstetric and critical-care resources, infection-prevention capacity, and the viral species and outbreak context.

## 4. Discussion

This review highlights the substantial burden of EVD during pregnancy while also demonstrating the complexity of interpreting the available evidence. As shown in Table 4, the literature consists largely of small retrospective outbreak reports, case series, and observational studies characterized by substantial heterogeneity in outbreak settings, viral species, diagnostic methods, and availability of supportive care. Similarly, the pooled estimates summarized in Table 6 reflect evidence derived from markedly different epidemiological and clinical contexts, which limit direct comparability across studies. Consequently, direct comparisons between historical and contemporary studies should be made with caution, and pooled estimates should not be interpreted as universally applicable across different epidemic contexts [33,47,48].

One of the most important findings emerging from the current evidence concerns maternal mortality. Early outbreak reports, particularly the Kikwit outbreak of 1995, described extremely high mortality among pregnant women and contributed to the longstanding perception that pregnancy is an independent predictor of death in EVD [10]. However, more recent evidence challenges this assumption. In the systematic review and meta-analysis by Kayem et al., the pooled maternal mortality rate was 67.8%, but substantial between-study heterogeneity was observed (I^2^ = 85%), and the relative risk of death among pregnant women compared with nonpregnant women was not significantly increased (RR 1.18, 95% CI 0.59–2.35) [47]. Similarly, data from the Mangina Ebola Treatment Center during the 2018–2020 Democratic Republic of the Congo outbreak demonstrated no increase in mortality among pregnant women after adjustment for important confounders, including age, viral load, vaccination status, and receipt of investigational therapeutics [48]. Collectively, these findings suggest that the historically reported excess mortality may have been influenced by limited supportive care, delayed diagnosis, and the absence of effective therapeutics rather than pregnancy alone. Nevertheless, EVD remains a life-threatening condition in pregnancy, and improved maternal survival should not be interpreted as evidence of reduced disease severity.

The differences between the historical studies summarized in Table 4 and the more contemporary cohorts suggest that improvements in supportive care, access to Ebola treatment centers, vaccination strategies, and investigational therapeutics may have contributed to improved maternal outcomes over time.

In contrast to maternal outcomes, fetal and neonatal outcomes remain overwhelmingly poor. The meta-analysis by Kayem et al. estimated a fetal loss rate of 76.9% and a neonatal mortality rate of 98.5%, emphasizing the devastating impact of EVD on pregnancy outcomes [47]. Importantly, fetal loss continued to occur frequently even in more recent cohorts where maternal survival improved substantially [48]. These observations suggest that advances in maternal care have not translated into comparable improvements in fetal and neonatal survival. The mechanisms underlying adverse fetal outcomes are likely multifactorial and may involve a combination of placental infection, impaired placental function, maternal hemodynamic instability, inflammatory dysregulation, coagulopathy, and direct fetal exposure to the virus. However, the relative contribution of each mechanism remains poorly understood because of the limited availability of detailed pathological and longitudinal studies.

Despite these advances, the findings summarized in both Table 4 and Table 6 indicate that fetal and neonatal outcomes remain disproportionately poor, highlighting a persistent gap between maternal and perinatal survival.

Another important contribution of this review is the clarification of terminology related to placental infection and vertical transmission. Previous reports have frequently interpreted the detection of Ebola viral RNA within placental tissues as evidence of vertical transmission. However, molecular detection alone does not establish productive viral replication, fetal infection, or the route by which neonatal infection may have occurred. The evidence reviewed here supports a hierarchical interpretation of transmission-related findings. Detection of viral RNA indicates the presence of viral genetic material, whereas immunohistochemical localization of viral antigen within placental cells provides stronger evidence of active placental infection. Muehlenbachs et al. demonstrated Ebola virus antigen within syncytiotrophoblasts and maternal placental mononuclear cells but not within fetal placental stromal cells or fetal organs, supporting placental involvement while simultaneously underscoring the limitations of equating placental infection with confirmed fetal infection [35]. Accordingly, placental viral detection, placental infection, fetal infection, and neonatal infection should be regarded as distinct entities requiring different levels of evidentiary support.

The evidence base for vaccination during pregnancy has expanded considerably since earlier reviews. Initial pregnancy-related data for rVSVΔG-ZEBOV-GP were derived primarily from inadvertent exposures and observational analyses involving relatively small numbers of pregnant women [52]. These studies provided valuable safety information but were limited in their ability to evaluate pregnancy-specific outcomes comprehensively. A major advance has been the publication of the Phase 3 trial of Ad26.ZEBOV/MVA-BN-Filo, which enrolled 2013 pregnant women and demonstrated reassuring maternal, fetal, and neonatal safety findings together with robust immunogenicity and passive transfer of antibodies to offspring [54]. These findings substantially strengthen the pregnancy-specific evidence base for Ebola vaccination. At the same time, it remains essential to distinguish between evidence of vaccine efficacy against Ebola virus disease, evidence of safety and immunogenicity during pregnancy, and policy recommendations governing vaccine use in pregnant populations. Furthermore, evidence generated for vaccines targeting Zaire ebolavirus should not be extrapolated automatically to other Ebola virus species, such as Bundibugyo virus, in the absence of supporting clinical data.

Compared with previous reviews, the principal value of the present review lies in its integration of emerging evidence across several interconnected domains. Specifically, it combines contemporary outcome data with recent advances in placental pathology, maternal–fetal transmission research, pregnancy-specific clinical-management guidance, and randomized vaccine evidence [33,46,47,48,54]. Rather than generating new pooled estimates, this review seeks to contextualize existing findings and to highlight areas where biological interpretation and clinical implications extend beyond simple mortality calculations.

Several limitations should be acknowledged. The available literature remains limited in both quantity and quality, with many studies consisting of case reports, small case series, or retrospective outbreak analyses. Variability in outcome definitions, diagnostic methods, follow-up duration, and reporting standards complicate comparisons across studies. The lack of standardized definitions for miscarriage, fetal death, stillbirth, and neonatal death across the available literature. Differences in gestational-age thresholds and outcome-reporting practices may contribute to the substantial heterogeneity observed among studies and complicate direct comparisons between outbreaks. In addition, overlapping outbreak cohorts may contribute to inadvertent double-counting if publications are interpreted independently rather than at the patient level. Although the present revision improves methodological transparency through enhanced search documentation, explicit study selection procedures, and detailed study-level synthesis, the conclusions remain constrained by the limitations of the underlying evidence base. Consequently, substantial knowledge gaps persist regarding the mechanisms of fetal injury, determinants of neonatal survival, long-term outcomes among exposed infants, and the optimal integration of vaccination and therapeutic strategies into pregnancy-specific care. Addressing these gaps will require prospective studies, standardized outcome reporting, and the continued inclusion of pregnant women in clinical research. Although Table 4 and Table 6 provide a structured synthesis of the available evidence, interpretation remains limited by small sample sizes, inconsistent outcome definitions, and possible overlap between outbreak reports.

## 5. Conclusions

EVD during pregnancy is associated with severe maternal disease and a particularly high burden of pregnancy loss and neonatal mortality. However, contemporary comparative studies do not clearly demonstrate that pregnancy independently increases maternal mortality, and historical estimates should be interpreted within their outbreak and treatment contexts. Evidence strongly supports involvement of the placenta and fetal compartments in EVD, but placental RNA detection, active placental infection, fetal infection, and neonatal infection are distinct evidentiary categories and should not be used interchangeably. Pregnancy-specific vaccine evidence has advanced substantially with randomized safety and immunogenicity data for Ad26.ZEBOV/MVA-BN-Filo, while observational evidence also informs rVSVΔG-ZEBOV-GP use. Clinical decisions require multidisciplinary care, rigorous infection prevention, and explicit recognition of areas where pregnancy-specific evidence remains very limited.

## Figures and Tables

**Table 1:** Selected major Ebola virus disease outbreaks since 1976. Data adapted from the CDC Ebola outbreak database.

Year(s)	Country/Region	Virus Species	Cases (n)	Deaths (CFR) ^1^	Notes
1976	Sudan	*Sudan virus*	284	151 (53%)	First recognized Sudan virus outbreak
1976	Democratic Republic of the Congo (formerly Zaire)	*Zaire ebolavirus*	318	280 (88%)	First recognized Ebola virus disease outbreak
1979	Sudan	*Sudan virus*	34	22 (65%)	Recurrent outbreak
1995	Democratic Republic of the Congo	*Zaire ebolavirus*	315	254 (81%)	Major outbreak in Kikwit
2000–2001	Uganda	*Sudan virus*	425	224 (53%)	Largest Sudan virus outbreak before 2022
2007	Uganda	*Bundibugyo virus*	131	42 (32%)	First identified Bundibugyo virus outbreak
2007	Democratic Republic of the Congo	*Zaire ebolavirus*	264	187 (71%)	Major Democratic Republic of the Congo outbreak
2012	Democratic Republic of the Congo	*Bundibugyo virus*	62	34 (55%)	Second major Bundibugyo virus outbreak
2014–2016	Guinea, Liberia, and Sierra Leone	*Zaire ebolavirus*	28,610	11,308 (39%)	Largest Ebola epidemic recorded
2018–2020	Eastern Democratic Republic of the Congo (with linked cases in Uganda)	*Zaire ebolavirus*	3470	2287 (66%)	Second largest Ebola virus disease outbreak in history
2022–2023	Uganda	*Sudan virus*	164	55 (34%)	Most recent Sudan virus outbreak

^1^ Abbreviations: CFR, case-fatality rate.

**Table 2:** Comparison of major Ebola virus species and implications for pregnancy.

Species	Virulence	Major Outbreaks	Vaccine Availability	Implications for Pregnancy
*Zaire ebolavirus* [12,13,14,17,18]	Highest	1976, 1995, 2014–2016, 2018–2020	rVSV-ZEBOV licensed	Severe maternal disease; extremely poor fetal outcomes
*Sudan ebolavirus* [10,15,18]	High	1976, 2000–2001, 2022–2023	None	High fetal loss; no vaccine
*Bundibugyo ebolavirus* [12,15,18,22]	Moderate	2007, 2012, 2026	None	Significant risk; major vaccine gap
*Taï Forest ebolavirus* [17,18]	Low	Rare	None	Limited data
*Reston ebolavirus* [17,18]	Non-pathogenic in humans	None	None	No known pregnancy impact

**Table 3:** Summary of maternal outcomes of Ebola virus disease during pregnancy.

Maternal Outcome	Evidence Summary	Key Considerations
Maternal mortality [11,33,50,51]	Historically extremely high; more recent outbreaks show CFR similar to non-pregnant women when advanced supportive care is available	Mortality driven by shock, multiorgan failure, and coagulopathy
Hemorrhage [20,39,40,41]	Vaginal bleeding, postpartum hemorrhage, DIC	Limited access to blood products increases risk
Multiorgan failure [29,30,41]	Common in severe disease	Major determinant of maternal death
Obstetric complications [10,33,41]	Miscarriage, preterm labor, PROM	Often occur regardless of maternal survival
Secondary infections [25,26,27]	Occurs due to immune dysregulation	Contribute to clinical deterioration

CFR = Case-Fatality Rate. Obstetric complications = including spontaneous miscarriage, intrauterine fetal demise, and preterm labor, are common and often occur regardless of maternal survival.

**Table 4:** Study-level characteristics and outcomes of selected primary studies.

Study/Outbreak	Design; Virus	Pregnant Women/Gestation	Treatment/Diagnostic Context	Maternal Outcome	Pregnancy/Neonatal Outcome	Key Limitation
Mupapa et al., 1999 [10]; Kikwit, DRC, 1995	Retrospective clinical series; Zaire ebolavirus outbreak	n = 15; 4 first, 6 s, 5 third trimester	Pregnancy based on history/clinical examination; historical supportive-care setting	1 survivor; severe bleeding reported in all 15	10 pregnancies ended in abortion; 3 Curettage; 1 premature stillbirth; 1 neonatal death;	Small historical cohort; retrospective; limited diagnostic/obstetric detail by modern standards
Baggi et al., 2014 [41]; Guinea, 2014	Two-case report; confirmed EVD	n = 2; the two reported at ~7 months;	RT-PCR confirmed; supportive care; induced vaginal delivery in both	Both women survived	Both fetuses died in utero, with high viral loads confirming massive intrauterine infection.	Case report; cannot estimate incidence or comparative risk
Philpott et al., 2023 [48]; Mangina ETC, DRC, 2018–2020	Observational cohort with propensity-score comparison; outbreak EVD	n = 27 pregnant women	ETC care; matching included age, initial viral load, vaccination, investigational therapy	No increased mortality vs. nonpregnant women: RR 1.0 (95% CI 0.58–1.72)	Among 19 surviving mothers, ≥11/19 (58%) pregnancy loss; 3/19 discharged with viable pregnancy; 2 live births, 1 infant survived	Single treatment center; small pregnancy sample; outcome data incomplete for some pregnancies

**Table 5:** Fetal and neonatal outcomes of Ebola virus disease during pregnancy.

Outcome	Frequency Reported	Evidence Summary
Miscarriage [10,33,41]	Very high across all trimesters	Often the first clinical manifestation
Intrauterine fetal demise [13,21,35]	Extremely common	Associated with placental infection and fetal hypoxia
Stillbirth [13,23,35]	The majority of third-trimester pregnancies	High viral load in the placenta and fetal tissues
Preterm birth [33,41]	Frequently reported	Often associated with maternal deterioration
Neonatal survival [10,33]	Rare	Only isolated case reports of survival

**Table 6:** Quantitative context from systematic syntheses.

Study	Evidence Base	Maternal Outcome	Fetal/Neonatal Outcome	Interpretive Caution
Foeller et al., 2020 [33]	Systematic review; 52 included studies	197/274 pregnant women with EVD died (72%); mortality not higher than general EVD population in the review	Nearly all women had adverse pregnancy outcomes; RNA persistence reported in amniotic fluid after blood clearance	Heterogeneous evidence including case reports and outbreak data; potential overlap across publications
Kayem et al., 2022 [47]	Systematic review/meta-analysis; 28 studies	Absolute maternal mortality 67.8% (95% CI 49.8–83.7; I^2^ = 85%); RR vs. nonpregnant 1.18 (95% CI 0.59–2.35)	Fetal loss 76.9% (95% CI 45.0–98.3; I^2^ = 96%); neonatal death 98.5% (95% CI 84.9–100)	Substantial heterogeneity; pooled estimates should not be treated as universally applicable

**Table 7:** Evidence for placental infection and vertical transmission of Ebola virus.

Tissue or Fluid	Viral Detection	Implications
Placenta [13,23,35]	High viral load	Major site of replication; immune-privileged reservoir
Amniotic fluid [13,35]	Frequently positive	Supports fetal exposure in utero
Fetal membranes [23,35]	Positive in multiple reports	Indicates widespread intrauterine contamination
Cord blood [21,35]	Positive	Strong evidence of vertical transmission
Fetal tissues [23,35]	Positive	Confirms in-utero infection

**Table 8:** Summary of Ebola vaccines and available evidence in pregnancy.

Vaccine/Regimen	Target/Evidence Context	Pregnancy-Specific Evidence	Interpretation for This Review
rVSVΔG-ZEBOV-GP (Ervebo)	Zaire ebolavirus; outbreak efficacy/effectiveness established outside pregnancy-specific efficacy studies	STRIVE: 84 peri-conception/early-pregnancy exposures; loss 14/31 immediate-vaccinated vs. 11/33 unvaccinated with known outcomes; no congenital anomalies among 44 live-born infants examined [52,53]	Pregnancy safety evidence is observational; do not equate population effectiveness with pregnancy-specific efficacy
Ad26.ZEBOV/MVA-BN-Filo	Heterologous two-dose Zaire-directed regimen	Phase 3 RCT in Rwanda: 2013 pregnant women randomized; no maternal/fetal or neonatal/infant safety concerns identified; robust binding-antibody responses and passive antibody transfer [54]	Direct pregnancy-specific randomized safety and immunogenicity evidence; not a pregnancy clinical-efficacy trial
Vaccines/candidates for Bundibugyo virus	Non-Zaire Ebola virus context	No pregnancy-specific efficacy evidence established in the sources synthesized here	Do not extrapolate protection from Zaire-directed vaccine evidence; describe use as investigational when applicable

## Data Availability

The original contributions presented in this study are included in the article/Supplementary Materials. Further inquiries can be directed to the corresponding author.

## Supplementary Materials

The following supporting information can be downloaded at: https://www.mdpi.com/article/10.3390/jcm15208169/s1, Table S1: Database search and study-selection reporting template.

## Abbreviations

The following abbreviations are used in this manuscript:

Abbreviation	Definition
rVSV-ZEBOV	Recombinant Vesicular Stomatitis Virus–Zaire Ebola Virus Vaccine (Ervebo^®^)
WHO	World Health Organization
CDC	Centres for Disease Control and Prevention
DRC	Democratic Republic of the Congo
CFR	Case-Fatality Rate
SANRA	Scale for the Assessment of Narrative Review Articles
RNA	Ribonucleic Acid
RT-PCR	Real-Time Reverse Transcription Polymerase Chain Reaction
DIC	Disseminated Intravascular Coagulation
IL-6/IL-8	Interleukin-6/Interleukin-8
TNF-α	Tumor Necrosis Factor-alpha
PROM	Premature Rupture of Membranes
IUFD	Intrauterine Fetal Demise
EVD	Ebola Virus Disease
EVD in pregnancy	Ebola Virus Disease occurring during pregnancy (contextual term)
Ervebo^®^	Trade name for rVSV-ZEBOV vaccine
Ad26.ZEBOV/MVA-BN-Filo	Heterologous prime-boost Ebola vaccine regimen
ChAd3-EBO	Chimpanzee Adenovirus Type 3 Ebola Vaccine Candidate
MeSH	Medical Subject Headings
PHEIC	Public Health Emergency of International Concern
