# Supplementary material for: Ebola Virus Disease in Pregnancy: Maternal–Fetal Outcomes, Vertical Transmission, and Implications for Vaccination—A Narrative Review
Source: J Clin Med. 2026 Oct 20;15(20):8169. doi: 10.3390/jcm15208169 (PMC13706550; doi:10.3390/jcm15208169)
Supplement: Supplementary file 1 [file jcm-15-08169-s001.zip › jcm-4589368-supplementary.pdf]

Supplementary Table S1. Database search and study-selection reporting template

Database	Exact search string	Last search date	Filters	Results retrieved (653)
PubMed (439)	[Ebola virus disease OR EVD] AND [pregnancy] AND [maternal outcomes]	16 May 2026	English	n = 38
	[Ebola OR EVD] AND [pregnancy] AND [fetal outcomes]	16 May 2026	English	n = 22
	[Ebola] AND fetal AND [vertical transmission]	16 May 2026	English	n = 11
	[Ebola] AND pregnancy AND [Vaccination OR Ebola vaccine]	16 May 2026	English	n = 368
Scopus (153)	(("Ebola virus disease" OR Ebola) AND (pregnancy OR pregnant) AND (placenta* OR neonatal OR vaccination))	15 May 2026	English	n = 153
Google Scholar (50)	Exact search terms	16 May 2026	None	Of 17800, the first 50 records were screened
WHO website (6)	Ebola Virus Disease in Pregnancy	16 May 2026	None	n = 6
CDC website (5)	Ebola Virus Disease in Pregnancy	16 May 2026	None	n = 5
